# Supplementary material for: Simultaneous functional MRI of two awake marmosets
Source: Nat Commun. 2021 Nov 16;12:6608. doi: 10.1038/s41467-021-26976-4 (PMC8595428; doi:10.1038/s41467-021-26976-4)
Supplement: Supplementary file 1 — Supplementary Information [file 41467_2021_26976_MOESM1_ESM.pdf]

Simultaneous functional MRI of two awake marmosets

Supplementary Methods

Eye movements during social interaction. To investigate the effects of social interactions on potentially linked patterns of eye movements between marmosets, we carried out eye movement recordings outside the scanner in an experimental setup approximating that used during MRI scanning. Marmosets M4 and M5 were head-fixed within the social coil and positioned facing each other at a viewing distance of 11 cm. At this distance, the face of the conspecific animal subtended a visual angle of approximately 20°. Eye movements were recorded at 500 Hz using video-oculography (EyeLink II, SR Research, Ottawa, ON, Canada). Separate cameras were placed in front of each animal out of the direct line-of-view of the conspecific animal.

To calibrate eye movements, marmosets viewed light-emitting diodes (LEDs), placed at known eccentricities, in the dark. A screen subtending 90° of visual angle was placed in front of the animals. Small LEDs (3.4°) were placed within the screen at an eccentricity of 24° to the left and right of centre and illuminated for a duration of 250 ms at an interstimulus interval of 3 s. The abrupt onset of these stimuli typically evoked saccades from the animals. This allowed the correlation between the voltage output of the eye tracker and the known eccentricity of the stimuli to be calibrated. The positions of the calibration stimuli were chosen such that they would roughly bracket the position of the conspecific animals' face. This procedure was carried out for each animal separately.

Following calibration, the room was re-illuminated, the screen was removed, and eye movements were recorded while the animals viewed each other for a duration of 15 minutes. Audio recordings of scanner sequences were played to simulate the MRI scanner environment.

Eye movement data was analyzed using custom python scripts. Calibrated eye traces were smoothed via linear convolution of an 11-sample-wide hamming window. A 20° square window was defined around the face of the conspecific animal's face; for each animal, it was determined when the animal's gaze was within this window. The degree of overlap between these time courses was then computed, with the process being subsequently repeated while shifting the time course of one animal forward or backward in time by one sample for up to 5 s. A null distribution was computed by randomly shuffling the eye position of one animal in time before computing the degree of overlap. This process was repeated 1,000 times and the 2.5th and 97.5th percentiles were determined at each time lag.

Assessing coil sensitivity dependence on angular position. The social coil is amenable to variable positioning within the scanner, including altering the coil's angle with $\mathbf{B_0}$. Since the coil's sensitivity to transverse magnetization is related to the transverse component of the receive field, $\mathbf{B_1}^-$, the angle of the coil with respect to $\mathbf{B_0}$ will alter the SNR profile. To quantify this dependency, a coil was loaded with a 3.8-cm-diameter spherical phantom, filled with 50-mM sodium chloride, and rotated through a range of angles with respect to $\mathbf{B_0}$ (0° to 90° in 5° increments). At each angle, a 3D gradient-recalled echo was acquired with and without RF transmission (matrix size: 224 × 92 × 64, FOV: 179 × 73 × 51.2 mm, TE/TR: 4.6/10 ms, flip angle: 20°, BW: 220 Hz/pixel, number of averages: 2). The covariance-weighted, root-sum-of-squares SNR was calculated[1, 2] at each angle using the complex data from individual elements.

Transmit flip-angle mapping. The transmit-field uniformity, as produced by the scanner's body coil, was measured to assess the difference in flip-angle between marmosets M3 and M4 when placed 11-cm apart and facing each other. Flip-angle maps were measured using a turbo, fast-low-angle-shot pulse sequence: matrix size: 256 × 80, FOV: 240 × 75 mm, number of slices: 12, slice thickness: 2 mm, TE/TR: 2.6/6,000 ms, flip angle: 8°, BW: 490 Hz/pixel. The mean flip angle over each head was computed in Matlab.

Supplementary coil performance metrics. Image SNR, receive sensitivity, and the geometry factor were measured as additional metrics to assess coil performance. For these analyses, marmosets M3 and M4 were placed 11-cm apart and facing each other, and a multi-slice, 3D gradient-recalled-echo image was acquired with and without RF transmission (matrix size: 288 × 104 × 64, FOV: 220 × 79 × 51.2 mm, TE/TR: 4.4/10 ms, flip angle: 20°, BW: 220 Hz/pixel, number of averages: 2).

Image SNR maps were derived from the complex images of individual elements using a covariance-weighted, root-sum-of-squares reconstruction[1, 2]. The constituent sensitivity profiles of receive elements were calculated by dividing the image of each receiver by the combined image.

The geometry factor was calculated by retrospectively under-sampling k-space by two-fold in the left-right direction—i.e., replicating the acceleration factor and field-of-view employed in the acquisition of functional images in this study. The worst-case geometry-factor was estimated by cropping the fully sampled images tight to the head prior to calculating the geometry factor in the left-right, superior-inferior, and anterior-posterior directions. Inverse geometry-factor maps were reconstructed in Matlab using the sensitivity encoding (SENSE) method[3].

Supplementary Discussion

Simultaneous eye tracking of two marmosets demonstrated that marmosets engaged in intermittent mutual eye gaze—i.e., a behaviour indicative of social interaction (Supplementary Fig. 3). The gaze time courses of marmosets M4 and M5 had peak overlap values at negative lags and lower overlap values at positive lags. This indicates marmoset M4 would look at the face of marmoset M5, followed by marmoset M5 looking at the face of marmoset M4, then the marmosets would break their mutual gaze. A similar pattern has been found in human-to-human gaze interactions and has been termed "antiphase synchrony"[4].

Image SNR followed the expected cosine dependency with angle to $\mathbf{B_0}$ (Supplementary Fig. 4). As the angle increases, the four lateral elements produce less transverse $\mathbf{B_1}^-$ and the coil element at the superior aspect of the head (which remains orthogonal to $\mathbf{B_0}$) begins to dominate the combined sensitivity profile. The largest decrease in image SNR, 18%, occurs at approximately 75°: the minimum does not occur at 90° because the normal vectors of the lateral elements are not orthogonal to the longitudinal axis of the coil. It is therefore recommended to position the two coils at conjugate angles to minimize their SNR disparity.

The social-coil method does not rely on a particular transmit coil; however, the transmit coil must be of sufficient size to produce a uniform and consistent flip angle across both marmosets, thereby obviating a commensurate variance in spatial and temporal SNR. Flip-angle maps acquired with the scanner's body coil (Supplementary Fig. 5) showed only a small difference in mean flip angle (5.8%) between marmosets M3 and M4 when placed 11-cm apart.

In vivo image SNR maps showed high SNR in the peripheral cortex, as expected for a surface coil array (Supplementary Fig. 6a). The difference in image SNR between marmoset M3 (in coil 1)

and marmoset M4 (in coil 2) was only 5% in the centre of the brain and 1% in the peripheral cortex. These differences are due to minor discrepancies between receive-array construction, marmoset anatomies, and transmit flip angle. The constituent sensitivity profiles of receive elements (Supplementary Fig. 6b) are spatially independent, which reduces the noise amplification during the reconstruction of accelerated images (i.e., the geometry factor).

The geometry factor was equal to unity throughout the brain when accelerating two-fold in the left-right direction with a field-of-view equivalent to that of functional acquisitions in this study. This was a result of the field-of-view in the left-right phase-encode direction being twice that of a marmoset's head width; therefore, image replicas created by under-sampling k-space did not overlap. After cropping images (prior to retrospective under-sampling) to ensure the maximum possible overlap of image replicas (i.e., the worst-case noise amplification), the mean/maximum geometry factor in the left-right, superior-inferior, and anterior-posterior directions was 1.30/1.79, 1.24/1.62, and 1.47/2.41, respectively (Supplementary Fig. 6c).

Supplementary Figures

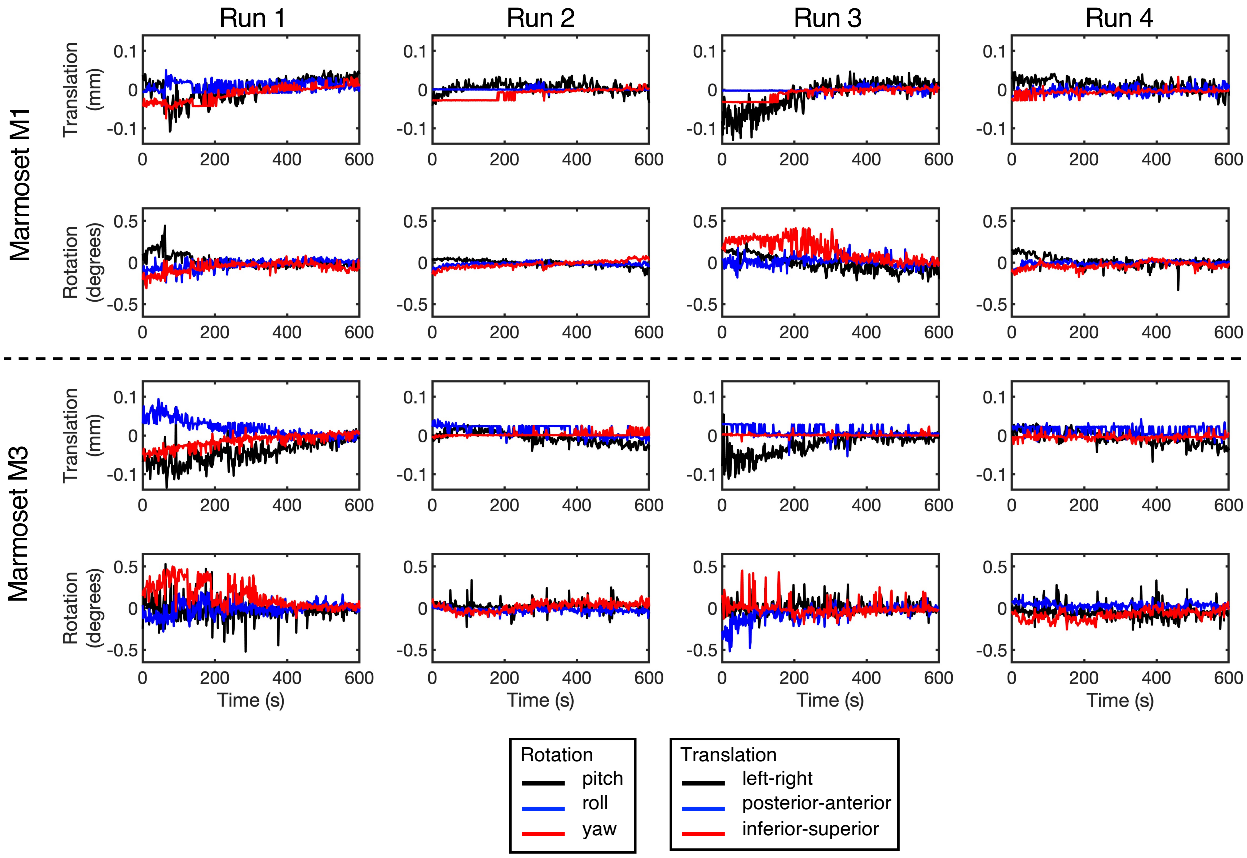

Supplementary Fig. 1 | Estimated motion during functional time courses. Translational and rotational motion of marmosets M1 and M3 during four, 10-min functional runs. The social coil employs four-point fixation of a chamber, resulting in less than 140 μm of translation and 0.6° of rotation during a single run.

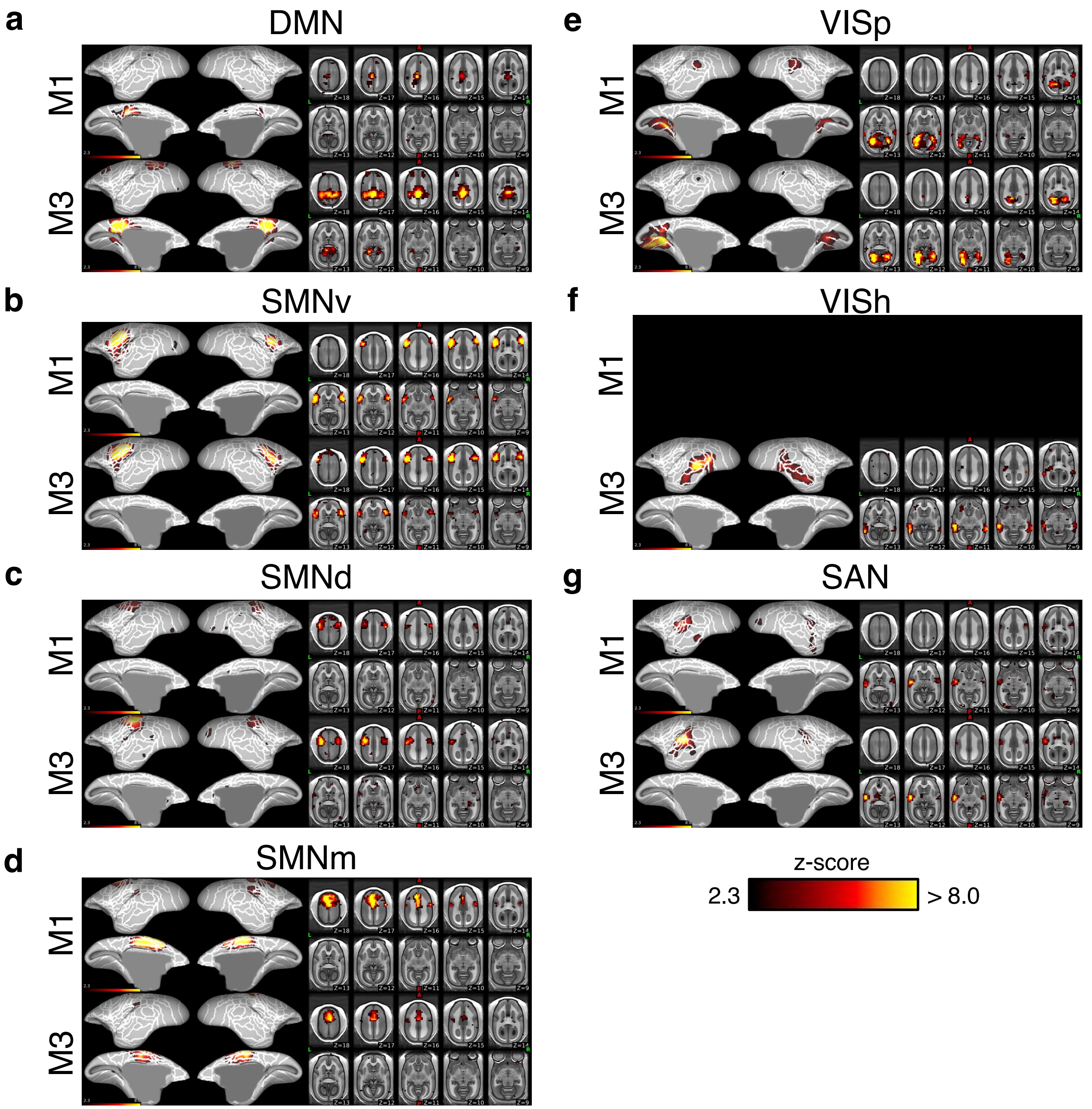

Supplementary Fig. 2 | Functional networks derived from the simultaneous scanning of two marmosets within each other's visual field. Functional networks surpassing the significance threshold were: **a**, default mode network (DMN); **b**, ventral somatomotor network (SMNv); **c**, dorsal SMN (SMNd); **d**, medial SMN (SMNm); **e**, primary visual network (VISp); **f**, high-order VIS (VISh); and **g**, salience network (SAN). These networks are presented as *z*-score maps on the template surface and volume. Connectivity maps had similar distributions between the two marmosets, although VISh did not meet the significance threshold for monkey M1. Improvements in *z*-score and network mapping can be achieved by averaging additional functional runs. White lines indicate cytoarchitectonic borders.

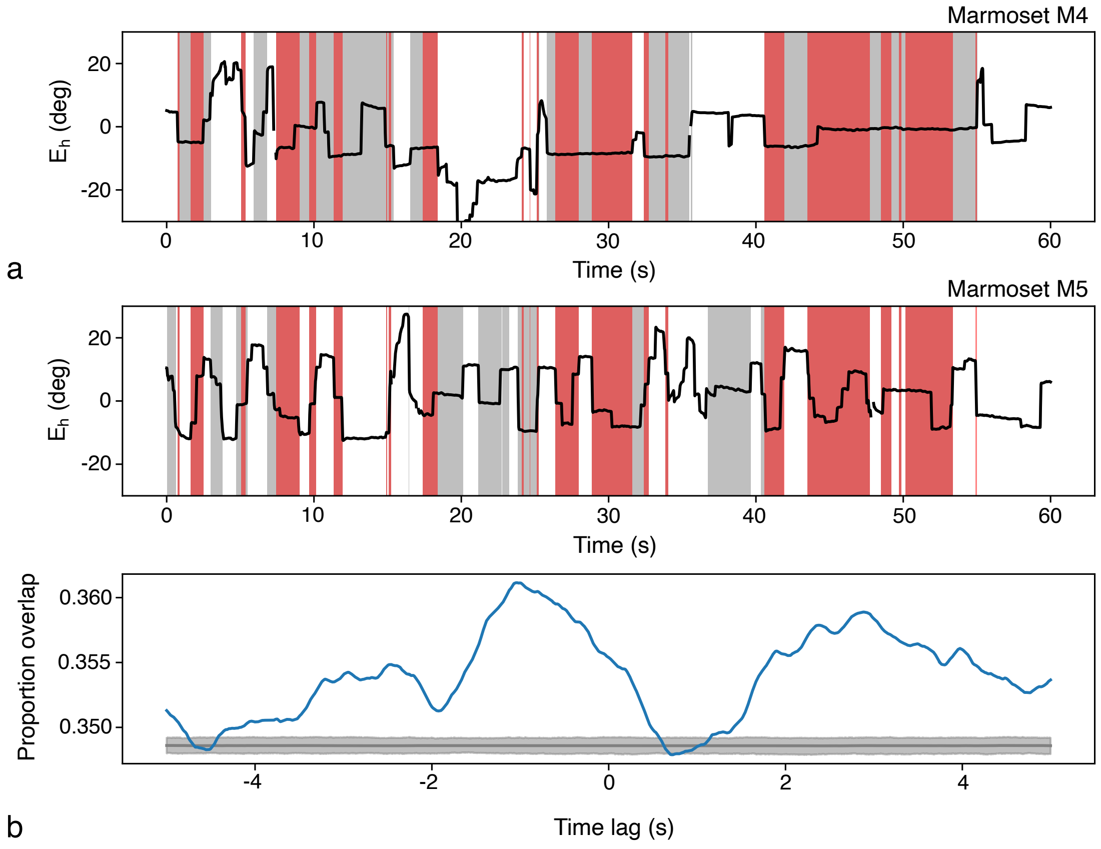

Supplementary Fig. 3 | Synchrony between marmoset gaze patterns. a, The horizontal gaze position, E_h, of two marmosets (M4 and M5) when viewing each other while head-fixed in the social coil. A representative 60-s excerpt is shown of the 15-minute recording session. Time spent looking at the conspecific marmoset's face is highlighted in grey for each marmoset and the overlap in this time between marmosets is highlighted in red. **b**, The proportion of time when gaze overlapped was computed at time lags ranging from -5 s to +5 s for the entire recording session. This indicates marmoset M4 would look at the face of marmoset M5, followed by marmoset M5 looking at the face of marmoset M4, then the marmosets would break their mutual gaze. A null distribution (grey line; range: 2.5^{th} – 97.5^{th} percentile; N: 1,000) was constructed by shuffling the eye position of one animal before computing the proportion of overlap at each time lag.

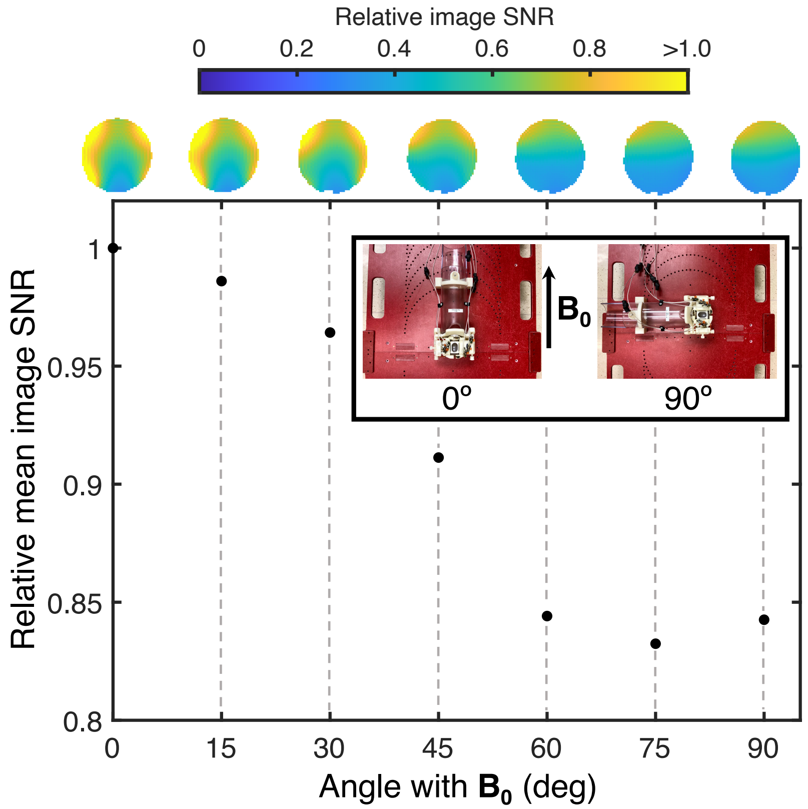

Supplementary Fig. 4 | Coil sensitivity dependence on angular position. The social coil can be positioned at varying angle to the longitudinal axis of the scanner to allow different physical arrangements for social interaction. Coil sensitivity to transverse magnetization (and therefore image SNR) is dependent on the angle of each element with $\mathbf{B_0}$. Mean image SNR, as a function of the coil positioning angle, is presented with the corresponding central transverse slice of the image SNR map. Lateral elements produce diminishing transverse $\mathbf{B_1}^-$ with increased angle; however, from Gauss's Law, the divergence of a magnetic field is zero (i.e., $\nabla \cdot \mathbf{B_1} = 0$); therefore, even when the normal vector of a coil element is parallel to $\mathbf{B_0}$, it will still have a transverse component (i.e., a non-zero sensitivity).

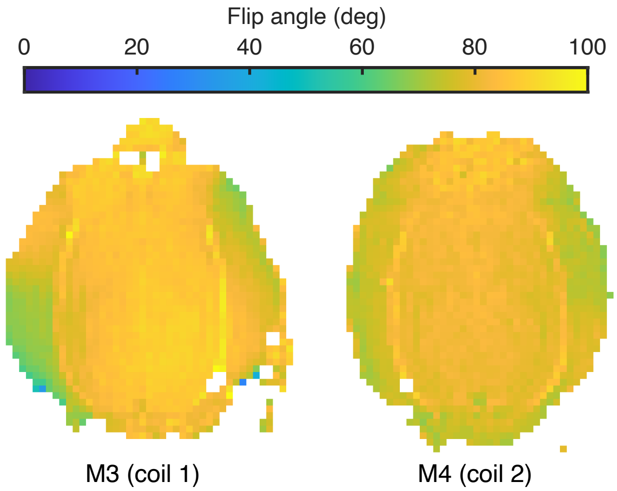

Supplementary Fig. 5 | Transmit flip-angle maps. A representative axial slice of a transmit flip-angle map acquired of marmosets M3 and M4 when placed 11-cm apart. The confluence of a large-diameter transmit body coil and the relatively small marmoset head produces a consistent flip angle between coils: the relative difference between mean flip angle of the phantoms differed by 5.8%. Flip-angle maps have been reoriented in radiological convention.

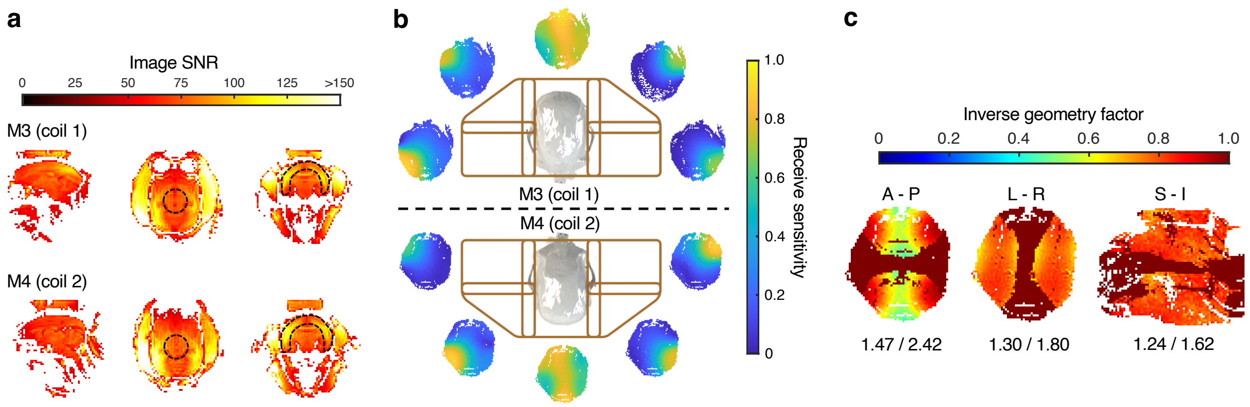

Supplementary Fig. 6 | Coil performance metrics. a, Image SNR maps, together with temporal SNR maps (Fig. 2), can be used to assess the similarity in performance between the two receive arrays. Representative sagittal, axial, and coronal slices show similar image SNR profiles between coils. Image SNR differs between the two receive array/marmoset combinations by 5% in the centre of the brain and 1% in the peripheral cortex (as depicted by the dashed ROIs). **b**, Receive sensitivity maps of individual receive elements (corresponding to the planar coil layout) govern the noise amplification during parallel-imaging reconstruction. Each receive array of the social-coil method is comprised of five elements—four laterally and one at the superior aspect of the head—thereby making it capable of a two-fold acceleration rate in each direction. **c**, Inverse geometry-factor maps in the anterior-posterior (A-P), left-right (L-R), and superior-inferior directions (S-I) were calculated with a two-fold acceleration rate and with images cropped to induce the worst-case geometry factor. The mean and maximum geometry factor are provided below individual maps.

Supplementary References

1. Roemer, P.B., Edelstein, W.A., Hayes, C.E., Souza, S.P. & Mueller, O.M. The NMR phased-array. *Magnetic Resonance in Medicine* **16**, 192-225 (1990).
2. Kellman, P. & McVeigh, E.R. Image reconstruction in SNR units: A general method for SNR measurement. *Magnetic Resonance in Medicine* **54**, 1439-1447 (2005).
3. Pruessmann, K.P., Weiger, M., Scheidegger, M.B. & Boesiger, P. SENSE: Sensitivity encoding for fast MRI. *Magnetic Resonance in Medicine* **42**, 952-962 (1999).
4. Tschacher, W., Tschacher, N. & Stukenbrock, A. Eye synchrony: A method to capture mutual and joint attention in social eye movements. *Nonlinear Dynamics Psychol Life Sci* **25**, 309-333 (2021).
