# Supplementary material for: Simultaneous functional MRI of two awake marmosets
Source: Nat Commun. 2021 Nov 16;12:6608. doi: 10.1038/s41467-021-26976-4 (PMC8595428; doi:10.1038/s41467-021-26976-4)
Supplement: Supplementary file 3 — Reporting Summary [file 41467_2021_26976_MOESM3_ESM.pdf]

nature research

Corresponding author(s): Kyle Gilbert

Last updated by author(s): Oct 15, 2021

Reporting Summary

Nature Research wishes to improve the reproducibility of the work that we publish. This form provides structure for consistency and transparency in reporting. For further information on Nature Research policies, see our Editorial Policies and the Editorial Policy Checklist.

Statistics

For all statistical analyses, confirm that the following items are present in the figure legend, table legend, main text, or Methods section.

n/a	Confirmed	
☐	☒	The exact sample size (*n*) for each experimental group/condition, given as a discrete number and unit of measurement
☐	☒	A statement on whether measurements were taken from distinct samples or whether the same sample was measured repeatedly
☐	☒	The statistical test(s) used AND whether they are one- or two-sided<br>*Only common tests should be described solely by name; describe more complex techniques in the Methods section.*
☒	☐	A description of all covariates tested
☒	☐	A description of any assumptions or corrections, such as tests of normality and adjustment for multiple comparisons
☐	☒	A full description of the statistical parameters including central tendency (e.g. means) or other basic estimates (e.g. regression coefficient) AND variation (e.g. standard deviation) or associated estimates of uncertainty (e.g. confidence intervals)
☐	☒	For null hypothesis testing, the test statistic (e.g. *F*, *t*, *r*) with confidence intervals, effect sizes, degrees of freedom and *P* value noted<br>*Give P values as exact values whenever suitable.*
☒	☐	For Bayesian analysis, information on the choice of priors and Markov chain Monte Carlo settings
☒	☐	For hierarchical and complex designs, identification of the appropriate level for tests and full reporting of outcomes
☒	☐	Estimates of effect sizes (e.g. Cohen's *d*, Pearson's *r*), indicating how they were calculated

Our web collection on statistics for biologists contains articles on many of the points above.

Software and code

Policy information about availability of computer code

Data collection	Python V3.4.2. Data collection code has been provided in a public OSF repository (DOI: 10.17605/OSF.IO/EJGF8).
Data analysis	For temporal-SNR analysis: Matlab R2019b and ImageJ 1.50a. For flip-angle, image SNR, receive sensitivity, and geometry factor analyses: Matlab R2019b. For motion estimates: Matlab R2019b and FSL V6.0.3. For continuous visual-conspecific analysis: FSL V6.0.3, Workbench V1.4.2, Matlab R2018b. For in-person versus pre-recorded video analysis: FSL V5.0.9, AFNI V18.0.11, Workbench V1.3.2. Computer-aided design files, image-processing pipelines, and a custom Matlab analysis tool have been provided in a public OSF repository (DOI: 10.17605/OSF.IO/EJGF8).

For manuscripts utilizing custom algorithms or software that are central to the research but not yet described in published literature, software must be made available to editors and reviewers. We strongly encourage code deposition in a community repository (e.g. GitHub). See the Nature Research guidelines for submitting code & software for further information.

Data

Policy information about availability of data

All manuscripts must include a data availability statement. This statement should provide the following information, where applicable:

- Accession codes, unique identifiers, or web links for publicly available datasets
- A list of figures that have associated raw data
- A description of any restrictions on data availability

Raw and preprocessed functional and anatomical data that support the findings of this study have been deposited in a public OSF repository (DOI: 10.17605/OSF.IO/EJGF8). Raw data has been provided for Figs. 2 – 6 and Supplementary Figs. 1,2,5, and 6.

Field-specific reporting

Please select the one below that is the best fit for your research. If you are not sure, read the appropriate sections before making your selection.

☒ Life sciences ☐ Behavioural & social sciences ☐ Ecological, evolutionary & environmental sciences

For a reference copy of the document with all sections, see nature.com/documents/nr-reporting-summary-flat.pdf

Life sciences study design

All studies must disclose on these points even when the disclosure is negative.

Sample size	The experimental setup of the social-coil design allowed for two animals to be scanned simultaneously, thus determining the sample size. All analyses were performed at a within-subject level or within subject-pair level to demonstrate the efficacy of the method.
Data exclusions	No data were excluded.
Replication	Experimental findings (brain activation maps) were acquired in two animals simultaneously, with similar results between animals, demonstrating reproducibility.
Randomization	Four marmosets were available for MRI scanning and were randomly paired for functional experiments.
Blinding	Blinding was not possible for this study, as animals had to be prepared for scanning before each experiment.

Reporting for specific materials, systems and methods

We require information from authors about some types of materials, experimental systems and methods used in many studies. Here, indicate whether each material, system or method listed is relevant to your study. If you are not sure if a list item applies to your research, read the appropriate section before selecting a response.

Materials & experimental systems

n/a	Involved in the study	
☒	☐	Antibodies
☒	☐	Eukaryotic cell lines
☒	☐	Palaeontology and archaeology
☐	☒	Animals and other organisms
☒	☐	Human research participants
☒	☐	Clinical data
☒	☐	Dual use research of concern

Methods

n/a	Involved in the study	
☒	☐	ChIP-seq
☒	☐	Flow cytometry
☐	☒	MRI-based neuroimaging

Animals and other organisms

Policy information about studies involving animals; ARRIVE guidelines recommended for reporting animal research

Laboratory animals	Imaging was performed on four common marmosets (Callithrix jacchus): 3-year-old males and a 2.5-year-old female.
Wild animals	The study did not involve wild animals.
Field-collected samples	The study did not involve samples collected from the field.
Ethics oversight	Experimental procedures were in accordance with the Canadian Council of Animal Care policy and a protocol (#2017-114) approved by the Animal Care Committee of the University of Western Ontario Council on Animal Care

Note that full information on the approval of the study protocol must also be provided in the manuscript.

Magnetic resonance imaging

Experimental design

Design type	Task and resting-state; block design.
Design specifications	Continuous visual-conspecific analysis data was acquired simultaneously of two marmosets for 4 runs, each consisting of 400 volumes. The total duration of each run was 10 minutes, with no time gaps between successive runs. In-person versus pre-recorded video data was acquired of a marmoset when either viewing a second marmoset in-person

(paradigm 1) or when viewing a pre-recorded video of the same marmoset (paradigm 2). Each paradigm consisted of 10 runs, with each run consisting of 172 volumes. Each run consisted of 17 alternating blocks: 18 s with opaque smart films between the two conspecifics (i.e., no visual contact) followed by 12 s with transparent smart films (i.e., with visual contact). The total duration of each run was 4 min 18 s. Approximately 30 s was required to set up successive runs.

Behavioral performance measures: During functional imaging, animals were monitored with a MRI-compatible camera to ensure they were not sleeping, thus ensuring they were completing their task of staying awake in order to view the other animal.

Acquisition

Imaging type(s): Functional and structural.

Field strength: 3T

Sequence & imaging parameters: The main sequence was a gradient-echo EPI. FOV: 220 x 78 mm, matrix size: 220 x 78, slice thickness: 1 mm, orientation: transverse, TE: 30 ms, TR: 1,500 ms, flip angle: 70 degrees.

Area of acquisition: A whole-brain scan was used.

Diffusion MRI ☐ Used ☒ Not used

Preprocessing

Preprocessing software: For continuous visual-conspecific analysis: FSL V6.0.3, Workbench V1.4.2, Matlab R2018b. For in-person versus pre-recorded video analysis: FSL V5.0.9, ANFI V18.0.11, Workbench V1.3.2.

Normalization: Non-linear normalization was performed to individual anatomical images (T1-weighted). This was followed by normalization to a T1-weighted marmoset template (https://marmosetbrainmapping.org/v3.html).

Normalization template: Normalization was initially performed to subject space and subsequently to a group standardized space (https://marmosetbrainmapping.org/v3.html).

Noise and artifact removal: ICA-based noise removal was performed for continuous visual-conspecific analysis. Motion correction and distortion correction were performed on both continuous visual-conspecific and in-person versus pre-recorded video data.

Volume censoring: Volume censoring was not performed.

Statistical modeling & inference

Model type and settings: Brain activation was estimated from individual subjects. A fixed-effect, two-sided t-test was used to compare the two conditions of a block design. An unpaired, voxel-wise t-test was used to compare the two paradigms' stimulus conditions.

Effect(s) tested: Brain activation maps were generated from EPI data, followed by a visual interpretation of preferentially active regions.

Specify type of analysis: ☒ Whole brain ☐ ROI-based ☐ Both

Statistic type for inference (See Eklund et al. 2016): All voxels were analyzed on a voxel-by-voxel basis.

Correction: To assess inter-brain correlated activity, correlation-coefficient maps were Fisher-Z transformed and masked to a threshold of 3.1.

Models & analysis

n/a	Involved in the study
☐	☒ Functional and/or effective connectivity
☒	☐ Graph analysis
☒	☐ Multivariate modeling or predictive analysis

Functional and/or effective connectivity: To assess inter-brain connectivity, correlation-coefficient maps were generated.
